# Safety and efficacy of a non-polymeric paclitaxel-eluting microporous stent in real-world percutaneous coronary intervention

**DOI:** 10.3892/etm.2013.1217

**Published:** 2013-07-11

**Authors:** SHAO-PENG WANG, RONG-CHONG HUANG, HAO ZHU, BO ZHANG, ZHEN-GUO ZHENG, DA YIN, JUN-JIE WANG, XU-CHEN ZHOU

**Affiliations:** Department of Cardiology, First Affiliated Hospital of Dalian Medical University, Dalian, Liaoning 116011, P.R. China

**Keywords:** coronary artery disease, percutaneous coronary intervention, YINYI™ stent, non-polymeric paclitaxel-eluting microporous stent, follow-up

## Abstract

At present, there is an increasing focus on stents that have a biodegradable polymer coating, rather than a permanent polymer coating. This is due to the fact that following the implantation of a drug-eluting stent (DES) with a permanent polymer coating, the continued existence of the coating may result in a foreign body reaction and delayed re-endothelialization. The aim of the present study was to evaluate the safety and efficacy of a non-polymeric paclitaxel-eluting microporous (YINYI™) stent in real-life percutaneous coronary intervention (PCI) for patients with coronary artery disease (CAD). A total of 686 YINYI™ stents were implanted in 404 patients with CAD in a PCI procedure and outpatient follow-ups were performed 1, 6, 12 and 15 months subsequent to the PCI, respectively. The observation endpoints were major adverse cardiac events (MACEs), including cardiac death, non-fatal myocardial infarction (MI), restenosis, target lesion revascularization, stent thrombosis and recurrence of angina pectoris. The average follow-up time was 15 months. The results revealed that the cumulative incidences of MACEs were as follows: mortality, 0.99%; non-fatal MI, 0.74%; restenosis, 4.0%; and target lesion revascularization, 2.7%. The results at the short- and long-term clinical follow-ups indicated that YINYI™ stents are effective and safe for use in PCI for patients with CAD.

## Introduction

Coronary artery disease (CAD) is a major mortality-causing disease, and percutaneous coronary intervention (PCI) is a conventional method for the treatment of CAD (1,2). The use of a bare metal stent (BMS) in PCI may reduce the incidence of restenosis for simple balloon dilatations; however, it is not completely accepted in clinical practice. Drug-eluting stents (DESs) improve the efficacy of percutaneous coronary artery revascularization by reducing neointimal development and the resultant in-stent restenosis (3,4). This improvement in PCI durability has led to the widespread adoption of DESs throughout the interventional cardiology community, while the appearance of first-generation DESs with permanent polymer coatings has resulted in a further reduction in the restenosis and target lesion revascularization rates compared with those of BMSs (5–7). PCI with DES implantation has become the standard of care for CAD, reducing the requirement for repeat intervention by 50–70% in comparison with BMSs. However, the follow-ups have revealed that the incidence of late and very late thrombosis of the DES following the discontinuation of dual antiplatelet drug administration increases progressively with time (8–10). It has been demonstrated that, following the implantation of a DES with a permanent polymer coating, the continued existence of the coating may result in a foreign body reaction and delayed re-endothelialization. This is one of the important pathogenic factors for late and very late thrombosis. At present, there is an increasing focus on stents that have a biodegradable polymer coating, rather than a permanent polymer coating. (11). In early selectively observed cases, it has been observed that a non-polymeric paclitaxel-eluting microporous (YINYI™) stent is safe and effective (12).

In the current study, the clinical application of a YINYI™ stent in real-world PCI was further investigated using a long-term follow-up, and the short- and long-term efficacy and safety of the stent were evaluated. Major adverse cardiac events (MACEs) were defined as mortality, non-fatal myocardial infarction (MI), restenosis, stent thrombosis, recurrence of angina pectoris and target lesion revascularization. It was observed that the incidence of MACEs was low and at an acceptable level. Furthermore, the number of stent thrombosis cases was reduced, in comparison with previous values for durable polymer coating stents. From the results of this study, it was concluded that the YINYI™ stent is safe and able to lower the incidence of stent thrombosis.

## Materials and methods

### Patients

Follow-ups were conducted on 404 patients from January 2008 to March 2010 in The First Affiliated Hospital of Dalian Medical University (Dalian, China). The average follow-up period was 15 months. All patients were implanted with YINYI™ stents (Liaoning Biomedical Materials R&D Center Co., Ltd., Dalian, China) in real-life PCI. This study was conducted in accordance with the Declaration of Helsinki and with approval from the Ethics Committee of The First Affiliated Hospital of Dalian Medical University. Written informed consent was obtained from all participants.

### PCI method

Following routine coronary angiography, the YINYI™ stent was implanted according to standard surgical procedures. The surgical approach for particular lesions was decided by the first surgeon. The lesion and balloon predilation sites were fully covered by the stent (releasing pressure >16 atm, 3–5 mm overlap for tandem stents). The criteria for a successful stent implantation comprised a satisfactory dilation and a residual stenosis rate of <20%. Multi-position projection, following nitroglycerin injection into the coronary artery, was performed to determine the pre- and postoperative degree of stenosis. Outpatient follow-ups were performed 1, 6, 12 and 15 months subsequent to the PCI, respectively.

### Peri- and postoperative medication

Prior to the surgery, aspirin (100 mg/day) and clopidogrel (75 mg/day) were orally administered for ≥5 days (for emergency surgery, 300 and 600 mg/day were administered, respectively). The postoperative aspirin dosage was adjusted to 300 mg/day, and, following three days, to 100 mg/day, while the clopidogrel dosage was adjusted to 75 mg/day. Tirofiban was selectively used according to the clinical condition of the patient.

### Follow-up endpoints and definitions

The follow-up endpoint was the occurrence of a MACE, defined as follows: i) Cardiac death, including unexplained sudden death; ii) non-fatal MI, where a new pathologic Q wave or ST-T change appeared in at least two adjacent leads in the electrocardiogram, where the creatine kinase (CK)-MB value was three-fold greater than the upper limit of the normal reference range or where the troponin level was five-fold greater than the normal reference range; iii) restenosis, where radiography revealed the stenosis rate of the target lesions in the stent implantation site and the segment 5 mm to the stent edge to be >70%; iv) stent thrombosis [Academic Research Consortium (ARC) definitions] (13), classified as definitive (where coronary angiography revealed a thrombus in the stent-implantation site and the segment 5 mm to the stent edge), possible (where unknown death occurred within 30 days postoperatively and MI occurred in the dominant region of the stent-implanted vessel at any time following the PCI) or suspicious (unexplained death 30 days following the PCI) stent thrombosis; v) recurrence of angina pectoris, where the angina pectoris reappeared for patients with a postoperative disappearance of the angina or returned to preoperative levels for patients with postoperative angina pectoris mitigation; vi) target lesion revascularization, where bypass surgery, stent re-implantation or balloon dilatation were required.

### Statistical analysis

Statistical analysis was performed using SPSS 11.0 statistical software (SPSS, Inc., Chicago, IL, USA). The data are expressed as the mean ± standard deviation, while the enumeration data are expressed as a frequency, with a 95% confidence interval. Multivariate Cox regression analysis was conducted to observe the correlation between the MACEs and the risk factors. P<0.05 was considered to indicate a statistically significant difference.

## Results

### General clinical data

A total of 404 patients were implanted with YINYITM stents from 2008 to 2010. The study population consisted of 350 males (86.6%) and 54 females (13.4%), with an average age of 63.5±10.3 years. One hundred and twelve patients (27.7%) presented with an acute MI, among whom 86 patients were treated with emergency stenting, while coronary angiography and stenting were conducted on the remaining 26. Hypertension was apparent as a complication in 62.6% of patients, and unstable or stable angina pectoris in 49% of patients. There were 94 cases with a history of recent or old MI (23.3%; Table I).

### Coronary angiography and PCI

As shown in Table II, there was a total of 598 target lesion sites. A total of 686 YINYI™ stents were implanted, which was an average of 1.70 stents per patient. There were 16 stents implanted in the left main coronary artery, 248 stents in the left anterior descending artery (12 stents in the diagonal branch of the artery), 164 stents in the left circumflex artery (68 stents in the obtuse marginal artery) and 258 stents in the right coronary artery (13 stents in the posterior descending artery). The site of stent implantation was selected according to the radiographic results. The lesions were classified as type A (43.8%), types B1 and B2 (40.1%, in combination) and complex type C lesions (16.1%; Table II). All patients satisfied the criteria of successful angiography. No recurrent ischemia, sudden death or MI occurred during hospitalization. The pressure of releasing stent was higher than that of other drug-eluting stents, and post-dilatation was necessary (Table III).

### Results of follow-ups

The results of the follow-ups are shown in Table IV. In the one-month follow-up, there were four fatalities (0.99%) due to sudden death and eight patients presented with recurrent angina pectoris, and were hospitalized for coronary angiography. Among these eight patients, stent restenosis was observed in three cases, resulting in target lesion revascularization treatment. The remaining five cases presented with mild intimal hyperplasia but not in-stent restenosis, and were treated with interventional therapy.

In the six-month follow-up, 13 cases (3.25%) presented with angina pectoris. Among these 13 cases, restenosis was observed in five cases (1.25%), three of which were treated with target lesion revascularization and two of which were re-hospitalized due to MI. There were no fatalities. The 12 month follow-up revealed 18 cases (4.50%) with a recurrence of angina pectoris, out of which restenosis was observed in eight cases (2%). Five of the eight cases were treated with target lesion revascularization, one case was treated with bypass surgery and one was re-hospitalized due to MI. In the 15 month follow-up, there was a recurrence of angina pectoris in 22 cases (5.5%). Among these 22 cases, restenosis was observed in 10 cases (2.5%), eight of which were treated with target lesion revascularization and one of which was treated with bypass surgery. No MIs or fatalities occurred during this follow-up period.

The cumulative incidence of MACEs was 8.17%. The incidences of cardiac death, non-fatal MI, restenosis, target lesion revascularization and recurrent angina pectoris were 0.99, 0.74, 6.44, 4.71 and 15.10%, respectively.

## Discussion

An ideal DES is required to possess a strong anti-restenosis ability and to effectively prevent late and very late thrombosis (14,15). The formation of stent thrombosis may be predominantly correlated with the polymer on surface of the DES (16). Therefore, for an ideal stent, there is a requirement for the polymer to be removed, resulting in a stent with the restenosis-reducing function of a DES and the long-term safety and clinical efficacy of a BMS (17,18). It has been observed in previous studies that the coating of a biodegradable drug-carrying polymer on the metal stent surface is able effectively inhibit intimal hyperplasia and negative remodeling following vascular injury, and reduce restenosis and late adverse reactions, such as stent thrombosis and late restenosis. The long term efficacy and safety of stents with biodegradable coatings has been indicated clinically (19–21).

Third-generation stents are ideal DESs; however, at present they are not frequently utilized clinically. The YINYI™ stent is made by microvias technology (Liaoning Biomedical Materials R&D Center Co., Ltd.), with special treatment of the metal surface, resulting in micro-blind holes that are able to store and carry drugs. This stent is a type of non-polymeric DES. The coverage rate of the microvia in the YINYI™ stent is >55%, with a shortening rate of <8%, a rebound rate of <10% and drug loading capability of 0.85±0.15 μg/mm^2^. It is a third-generation DES currently used in clinical practice worldwide.

In this study, YINYI™ stents were used in real-life PCIs for various types of CADs and lesions. With regard to the disease type, angina pectoris accounted for 49% of total cases and a history of old or recent MI and acute MI accounted for 23.3 and 27.7%, respectively. With regard to the lesion type, as observed by coronary angiography, the diseases included various types of lesions, such as left main coronary artery, bifurcation, chronic occlusive and long lesions. The proportion of each type of lesion was identical to that observed in general clinical practice, with good imageology and long-term follow-up results.

Hannan *et al*(12) observed that the mortality rate within one month subsequent to the PCI was ~0.94% (hospitalization, 0.56%; discharge, 0.38%). The majority of the mortalities were young patients with a more efficient heart function. In the current study, there were four (0.99%) fatalities within one month subsequent to the PCI, which was similar to the result of the study by Hannan *et al*. The predominant cause of mortality was sudden death, which was correlated with stent thrombosis (ARC definition). There have been no fatalities within the treatment group since 2009. The results of the long-term clinical follow-up revealed that the restenosis rate for the YINYI™ stent within two and six months postoperatively was 4 and 1.25%, respectively. This was similar to the results obtained in previous studies (22,23), which indicated that the early restenosis rate was 5% and that the restenosis rate in the clinical follow-up was relatively low.

According to the basic structural characteristics of the YINYI™ stent, there is a requirement for the stent to be released under high pressure, in order that the drug-carring suface is in close proximity to the vascular intima. This ensures the slow-release of the drug over an extended period of time. At present, the releasing pressure of a DES with permanent polymer coating is ~6 atm, with a low ratio of high-pressure post-dilatation. In this study, the average releasing pressure of the YINYI™ stent was 14 atm, with a higher ratio of high-pressure post-dilatation. The limitation of this study was the relatively small sample size and a further study of the clinical results from a five year follow-up is required. Moreover, the sample size for complex lesions in the present study was small, due to the limitation of stent length, and this therefore requires further investigation.

In conclusion, the YINYI™ stent has been demonstrated in short and long-term clinical follow-ups to be effective and safe for use in PCI for patients with CAD.

## Figures and Tables

**Table I:** General clinical data for patients implanted with a non-polymeric paclitaxel-eluting microporous (YINYI™) stent (n=404).

Status	Value	Percentage (%)
Male (n)	350	86.6
Female (n)	54	13.4
Age (years)	63.5±10.3	-
Smoking (n)	176	43.6
Family history (n)	56	13.9
Hypertension (n)	253	62.6
Diabetes mellitus (n)	143	35.4
Angina pectoris (n)	198	49.0
Cerebrovascular disease (n)	103	25.5
Acute myocardial infarction (n)	112	27.7
Hyperlipemia (n)	154	38.1
Recent/old myocardial infarction (n)	94	23.3

The value for age is expressed as the mean ± standard deviation.

**Table II:** Results of coronary angiography and percutaneous coronary intervention.

Variable	Number	Percentage (%)
Lesion site (n=598)		
Left anterior descending artery	209	34.9
Left circumflex artery	150	25.1
Right coronary	223	37.3
Left main coronary artery	16	2.7
Lesion type		
Type A	262	43.8
Type B1	118	19.7
Type B2	122	20.4
Type C	96	16.1
Complex lesions (n=96)		
Left main coronary artery	16	2.7
Long lesion (≥30 mm)	27	9.7
Chronic occlusion	12	2.0
Ostial lesion	9	1.5
Small vessel (diameter ≤2.7 mm)	24	5.0
Bifurcation lesion	9	2.8
Calcification and angulation	11	2.0
Number of stents (n=686)		
Left main coronary artery	16	2.3
Left anterior descending artery	248	36.2
Diagonal branch artery	12	1.7
Left circumflex artery	164	23.9
Obtuse marginal artery	68	9.9
Right coronary	258	37.7
Posterior collateral coronary and posterior descending artery	13	0.2

**Table III:** High-pressure post-dilatation.

Lesions (n)	Releasing pressure (atm)	High-pressure post-dilatation [n (%)]
Left main coronary artery (16)	20	20 (100)
Left anterior descending artery (248)	16	199 (80.2)
Left circumflex artery (150)	14	95 (83.3)
Right coronary (223)	16	124 (55.6)
Long lesion (≥30 mm) (58)	18	58 (100)
Chronic occlusion (12)	16	10 (83.3)
Ostial lesion (9)	20	9 (100)
Small vessel (diameter ≤2.7 mm) (30)	14	23 (76.7)
Bifurcation lesion (17)	16	17 (100)
Calcification and angulation (12)	18	12 (100)

**Table IV:** Incidences of major adverse cardiac events (MACEs) at follow-ups of 1–15 months.

MACE	1 month n (%)	6 months n (%)	12 months n (%)	15 months n (%)	Cumulative incidence (%)
Primary endpoints					
Cardiac death	4 (0.99)	0 (0.00)	0 (0.00)	0 (0.00)	0.99
Restenosis	3 (0.74)	5 (1.25)	8 (2.00)	10 (2.50)	6.44
Non-fatal myocardial infarction	0 (0.00)	2 (0.50)	1 (0.25)	0 (0.00)	0.74
Secondary endpoints					
Target lesion revascularization	3 (0.74)	3 (0.75)	5 (1.25)	8 (2.00)	4.71
Bypass surgery	0 (0.00)	0 (0.00)	1 (0.25)	1 (0.25)	0.50
Recurrence of angina pectoris	8 (1.98)	13 (3.25)	18 (4.50)	22 (5.50)	15.10

At 1 month, n=404; at 6, 12 and 15 months, n=400.
